# The “Menopause” knockout; a science-backed therapeutic ally, and strategy for midlife wellness

**DOI:** 10.3389/fspor.2025.1682887

**Published:** 2025-12-18

**Authors:** Gayathri Delanerolle, Peter Phiri, Sohier Elneil

**Affiliations:** 1 Institutes of Applied Health Research, University of Birmingham, Birmingham, United Kingdom; 2 Clinical Trials Facility, Hampshire and Isle of Wight Healthcare NHS Foundation Trust, Southampton, United Kingdom; 3 School of Medicine, University of Southampton, Southampton, United Kingdom; 4 Institute of Womens Health, University College London, London, United Kingdom

**Keywords:** menopause, boxing, science-Backed therapeutic ally, strategy for midlife wellness, women

## Abstract

Boxing, traditionally a competitive sport, is increasingly recognised as a therapeutic exercise modality for women transitioning through perimenopause and menopause a life stage characterised by hormonal changes that can accelerate muscle loss, bone demineralisation, balance impairment, cardiovascular risk, weight gain, and mood fluctuations. Structured, non-contact fitness boxing integrates resistance, impact, and aerobic components, delivering multi-system benefits relevant to this population. Physically, boxing stimulates muscle protein synthesis, preserves lean mass, and provides weight-bearing stimuli to maintain bone density, thereby reducing fracture risk. Dynamic footwork and agility drills challenge proprioception and postural control, improving balance and lowering fall risk. The high-intensity cardiovascular demands enhance heart health, reduce blood pressure, improve lipid profiles, and assist with weight management. Physiologically, boxing's combined strength-endurance format boosts basal metabolic rate, improves insulin sensitivity, and moderates stress hormone levels, supporting metabolic health and resilience to menopause-related changes. Neuromuscular adaptations from complex motor sequences enhance coordination, reaction time, and functional independence. Neurologically, boxing promotes endorphin release and modulates key neurotransmitters such as serotonin and dopamine, improving mood stability and reducing anxiety. Cognitive engagement through learning and executing punch combinations enhances brain-derived neurotrophic factor (BDNF) levels, supporting neuroplasticity, memory, and executive function. Emerging evidence positions non-contact boxing as a safe, engaging, and multi-dimensional exercise strategy for midlife women. It addresses physical, physiological, and neurocognitive domains in one intervention, offering healthcare and wellness professionals a practical, evidence-informed tool to promote strength, stability, cardiovascular fitness, and psychological well-being during the menopausal transition.

## Introduction

Menopause is a biological transition characterised by the decline of ovarian hormone production, most notably oestrogen and progesterone. These hormonal changes generate a wide range of physiological, neurochemical, and psychosocial effects, including vasomotor symptoms, alterations in mood and cognition, changes in body composition, reduced bone density, impaired balance, sleep disturbances, and increased cardiometabolic risk (1). For many women, these symptoms intersect with midlife responsibilities, work pressures, and social expectations, compounding their overall impact on health and well-being.

There is evidence suggesting exercise plays an important role in alleviating many menopause-related symptoms (2). Aerobic exercise supports cardiovascular health, weight management, and thermoregulation; resistance training improves bone density, muscle mass, and metabolic function; and neuromotor activities can enhance balance, coordination, and cognitive performance. Collectively, exercise interventions have been shown to improve mood, reduce anxiety, enhance sleep quality, and support overall quality of life during the menopausal transition (3).

Boxing is traditionally a competitive sport. It is increasingly recognised as an effective form of exercise therapy for women navigating perimenopause and menopause. This life stage is marked by hormonal shifts that can lead to muscle loss, reduced bone density, balance impairments, cardiovascular changes, weight gain, and mood fluctuations. A structured boxing training programme focusing on non-contact fitness boxing can counteract many of these issues. The evidence-informed physical, physiological, and neurochemical benefits of boxing for women in midlife, in a formal analysis suitable for healthcare and wellness professionals.

**Figure 1:**
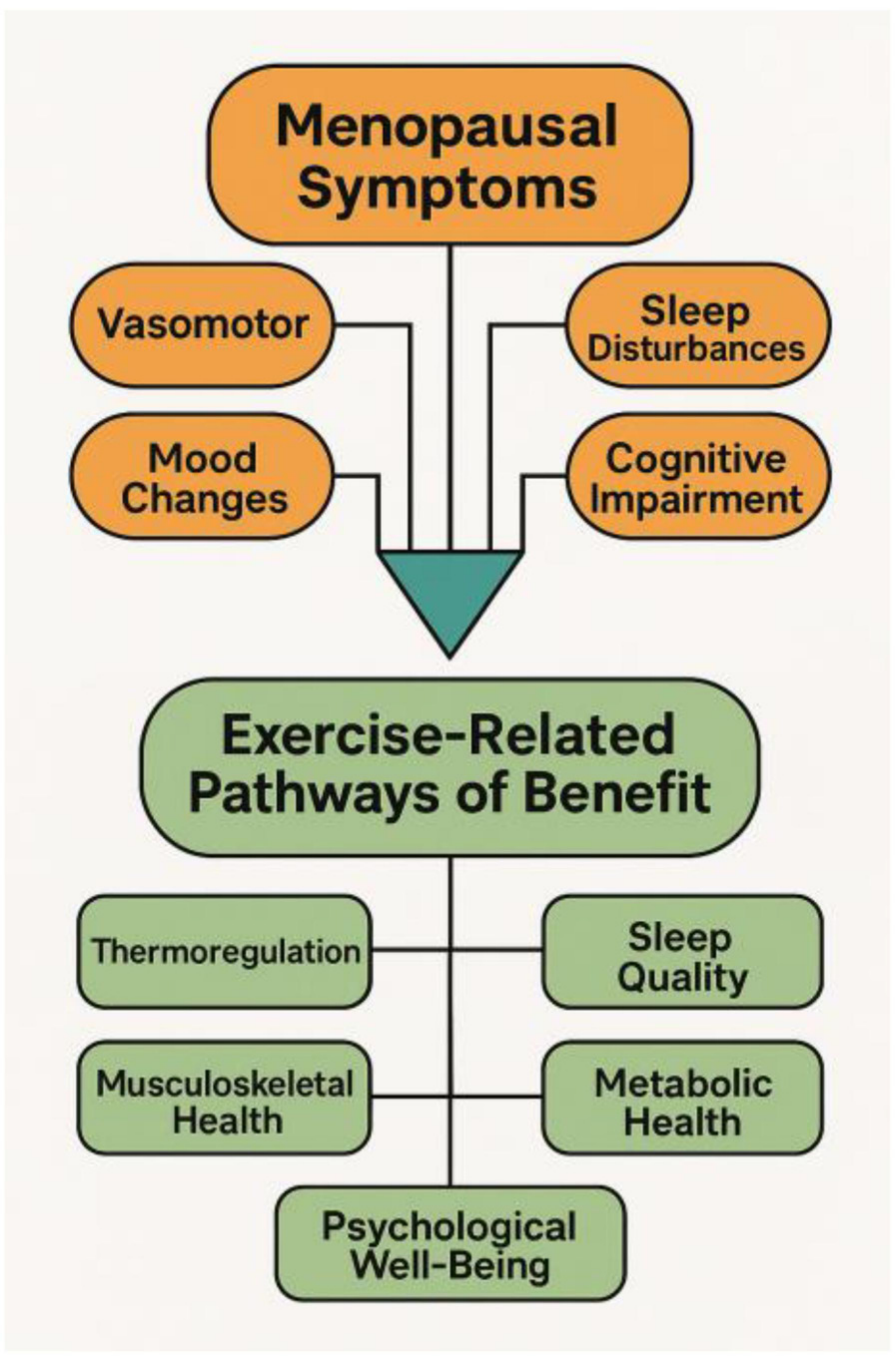
Menopausal symptoms and exercise-related pathways of benefit schematic overview illustrating key menopausal symptom domains (vasomotor, sleep, cognitive, and mood-related) and the primary physiological and psychological pathways through which exercise may alleviate these symptoms. The diagram highlights how common menopausal changes connect to evidence-based exercise-mediated mechanisms that support health and well-being.

Boxing warrants specific attention because it integrates multiple exercise modalities within a single activity where high-intensity interval style cardiovascular work, resistance-based movements, neuromotor and coordination challenges, and substantial cognitive engagement (4). Its combination of footwork, reactive movement, sequencing, and upper- and lower-body power output provides a unique blend of physiological and psychological stimulation that aligns directly with many of the domains affected by menopause. Additionally, the neurochemical responses associated with boxing such as endorphin release, improved dopamine regulation, and reductions in stress-related cortisol may offer further benefits for mood stability and cognitive clarity (Figure 1).

Boxing is traditionally a competitive sport. It is increasingly recognised as an effective form of exercise therapy for women navigating perimenopause and menopause. This life stage is marked by hormonal shifts that can lead to muscle loss, reduced bone density, balance impairments, cardiovascular changes, weight gain, and mood fluctuations. A structured boxing training program focusing on non-contact fitness boxing can counteract many of these issues. The evidence-informed physical, physiological, and neurochemical benefits of boxing for women in midlife, in a formal analysis suitable for healthcare and wellness professionals.

## Physical effects of boxing in midlife women

Muscle strength and bone density is an important consideration during menopause as oestrogen decline accelerates sarcopenia and osteoporosis. Boxing provides resistance and impact stimuli that build muscular strength and help maintain bone mass. The vigorous punching and footwork engage major muscle groups, stimulating protein synthesis and neuromuscular adaptation to preserve muscle function (5). Stronger muscles in turn protect joints and support the skeletal system. High-impact, weight-bearing exercise such as boxing can slow bone loss and even increase bone density, which is crucial to counteract menopause-associated bone demineralisation (6). Indeed, early evidence links boxing training to increased bone density in menopausal women, helping fight off osteoporosis and improving overall physical function. By preserving lean muscle and bone strength, boxing training supports functional independence and reduces fracture risk in this population (7).

Balance often worsens with age and estrogen loss, increasing fall risk. The dynamic movements in boxing including shifts in stance, directional changes, and coordinated punches challenge the body's balance systems. Over time, this improves proprioception, core stability, and postural control (8). Research shows that targeted exercise interventions can significantly improve both static and dynamic balance in perimenopausal and postmenopausal women. In practice, boxing's complex footwork and agility drills act as balance training, potentially reducing fall and injury risk. Maintaining neuromotor fitness via such complex motor skills is imperative in older age; it not only reduces falls but also helps maintain physical and cognitive functioning. Thus, boxing serves as a functional balance exercise, enhancing stability and coordination (9).

Boxing is a high-intensity aerobic workout that elevates heart rate and improves cardiovascular endurance. Rounds of boxing drills provide vigorous cardio stress that strengthens the heart muscle and improves circulation. This is especially beneficial as menopause is associated with a higher risk of cardiovascular disease (10). Regular boxing workouts can help lower blood pressure and improve lipid profiles, contributing to heart health. Additionally, boxing's calorie-intensive nature aids in weight management. Menopausal women often experience a slowing metabolism and increased fat accumulation where boxing could be a mitigatory approach (11). The intensive cardio and strength components raise the metabolic rate, helping to replace fat with muscle and prevent menopause-related weight gain. Increasing lean muscle through boxing further boosts resting metabolic expenditure, countering the typical post-menopausal metabolic decline. Consistent training thus assists with healthy weight control and body composition, which in turn reduces risks of metabolic disorders (12).

## Physiological impacts on metabolism and hormonal balance

Engaging in boxing yields significant metabolic benefits. As noted, adding lean muscle mass elevates basal metabolic rate, meaning the body burns more calories at rest. This helps offset the drop in metabolism that often comes with menopause, thereby aiding in weight control and energy levels. Importantly, boxing combines aerobic and resistance elements that improves insulin sensitivity (13). Improved insulin sensitivity facilitates better blood glucose control and can reduce the risk of insulin resistance and metabolic syndrome in midlife women. High-intensity interval aspects of boxing training also promote favourable lipid profiles and cardiovascular conditioning, further supporting metabolic health (14).

While boxing cannot replace diminished estrogen, it can favourably influence other hormonal pathways. Exercise moderates stress hormones: regular physical activity is shown to reduce circulating cortisol and adrenaline levels, which tend to be elevated under chronic stress. By lowering these stress hormones, boxing may alleviate menopausal symptoms like anxiety and sleep disturbances. Concurrently, intense exercise triggers the release of anabolic hormones and growth factors (15). Studies on postmenopausal women have found that structured strength training which boxing emulates in many ways can improve the hormonal profile. For example, elevating growth hormone and IGF-1 levels that support tissue health. A recent systematic review concluded that exercise performed 2–3 times per week for several months led to significant improvements in muscle strength, bone density, and hormonal and metabolic markers in menopausal women compared to inactive controls (16). These findings suggest boxing, as a form of strength-endurance training, helps stabilise physiological changes. Notably, exercise may also reduce vasomotor symptoms by enhancing thermoregulatory control and improving vascular function. Overall, boxing promotes a more favourable hormonal balance and physiological resilience during menopause (17).

Boxing requires synchronising complex movements coordinating punches with footwork and defensive actions. This continuous training of neuromuscular coordination yields significant benefits. Each punch involves rapid activation of muscle fibbers and precise timing, which strengthens neural pathways between the brain and muscles. Over time, boxing practice enhances reaction time, coordination, and agility. Such neuromuscular adaptation is critical in countering age-related declines in coordination (18). Resistance exercises are known to preserve neuromuscular function by driving adaptations in motor unit recruitment and efficiency. Moreover, participating in complex motor skill activities has been shown to improve neuromotor fitness, which is linked to better balance and even cognitive maintenance in older adults. In essence, boxing serves as both a physical and neural workout, sharpening the body's coordination and reflexes which might otherwise dull with menopause and aging (18).

## Effects on brain chemistry and cognitive function

One of the most immediate benefits of boxing exercise is the release of endorphins-neuropeptides that act as natural painkillers and mood elevators. Sustained moderate-to-high intensity exercise like boxing triggers the brain to increase endorphin production (19). These “feel-good” hormones bind to opioid receptors in the brain, reducing the perception of pain and inducing feelings of euphoria and stress relief. Many women in perimenopause experience mood swings, anxiety, or mild depression (1). The endorphin surge from a boxing workout can acutely improve mood and promote a sense of well-being. In fact, higher-intensity workouts are associated with a greater endorphin response than mild exercise. By regularly engaging in boxing, women may harness these neurochemical benefits to help regulate mood and cope with menopausal stressors in a healthy way (20).

**Figure 2:**
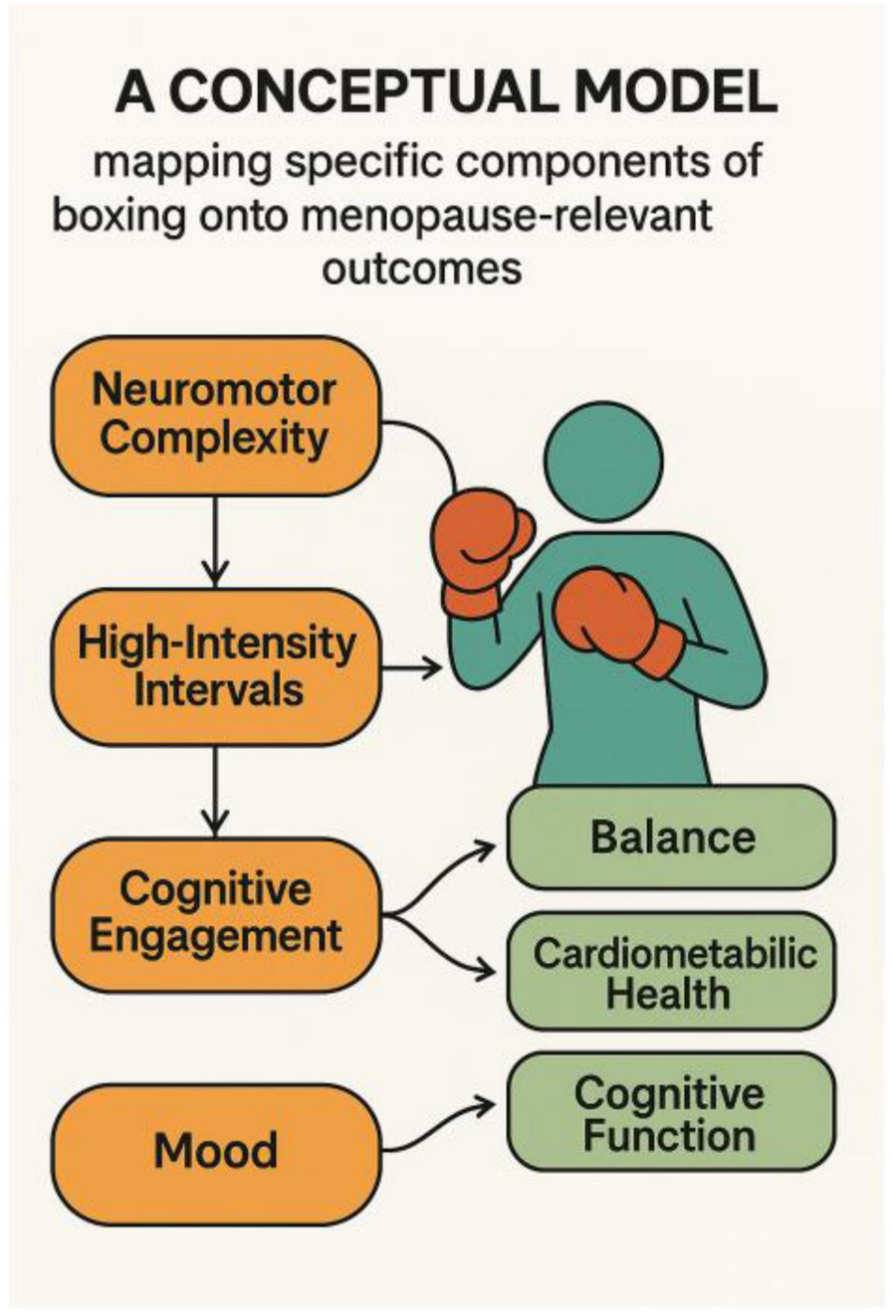
Conceptual model linking boxing components to menopause outcomes conceptual model mapping core components of boxing, neuromotor complexity, high-intensity intervals, cognitive engagement, and mood enhancement to menopause-relevant outcomes. The figure shows how the multimodal nature of boxing aligns with benefits for balance, cardiometabolic health, mood regulation, and cognitive function in midlife and menopausal women.

Beyond endorphins, exercise modulates other neurotransmitters that affect emotional and cognitive health. Physical activity has been shown to increase levels of serotonin and dopamine in the brain. Serotonin is crucial for mood stability and is often called the “feel-good” neurotransmitter, while dopamine is involved in reward, motivation, and executive function. Enhanced serotonin and dopamine release through exercise can mimic some effects of antidepressant therapies, contributing to reduced depressive symptoms and anxiety in physically active individuals. At the same time, exercise can decrease excess excitatory neurotransmitters like glutamate that are linked with anxiety and stress responses. The result is a more balanced neurotransmitter environment: higher serotonin and dopamine promote positive mood and cognitive focus, whereas lower chronic glutamate and cortisol reduce anxiety (20). Clinically, regular exercise is associated with lower rates of depression and improved stress resilience in midlife, partly due to these neurochemical adjustments. For menopausal women, who may face increased risk of mood disturbances, boxing could offer a drug-free method to support neurochemical balance and mental health (Figure 2).

Engaging in boxing can also confer cognitive benefits, thanks to both the physical exercise itself and the mental demands of the sport. Aerobic exercise is well known to stimulate the release of neurotrophic factors like brain-derived neurotrophic factor (BDNF), which support neuron growth and survival, enhancing brain plasticity. Higher BDNF levels are associated with better memory and learning, and exercise-induced BDNF may help counteract the “brain fog” sometimes reported during menopause. In addition, the cognitive challenge of learning punch combinations and defensive techniques exercises the brain (1, 21, 22). Boxing is a cognitively engaging activity requiring concentration, quick decision-making, and motor learning. Such complex exercise has been linked to improvements in executive functions (like multi-tasking and decision speed) and may protect against cognitive decline (23). In resistance training studies, participants showed not only physical improvements but also enhanced cognitive performance, likely through improved neuroplasticity and cerebral blood flow. Thus, boxing's combination of physical exertion and mental strategy uniquely positions it to boost cognitive health. Over time, consistent training can improve reaction time, sharpen attentional focus, and bolster memory through repeated practice of complex motor sequences. This cognitive stimulation, paired with the neurochemical benefits of exercise, makes boxing a holistic brain training modality for women in midlife (24, 25).

Boxing is emerging as a comprehensive physical therapy strategy for perimenopausal and menopausal women, addressing multiple health dimensions in one modality. Physically, boxing training builds muscle strength, preserves bone density, improves balance, enhances cardiovascular fitness, and aids in weight management. Physiologically, it boosts metabolic function and moderates hormonal changes improving insulin sensitivity, reducing stress hormones, and supporting neuromuscular coordination. Neurologically, boxing exercise favourably alters brain chemistry by releasing endorphins and regulating neurotransmitters, while the mental engagement in complex boxing techniques promotes cognitive resilience. All of these benefits are supported by a growing body of evidence in exercise science and menopause health. For clinicians and wellness practitioners, boxing (when properly supervised to ensure safety) can be recommended as an effective, multi-faceted exercise intervention to help midlife women maintain their physical health and psychological well-being through the menopausal transition. The integration of boxing into wellness programs offers an engaging way to empower women, helping them stay strong in body and mind during this life stage.

## Data Availability

The original contributions presented in the study are included in the article/Supplementary Material, further inquiries can be directed to the corresponding author.
